# Comparison of Virtual Patient Simulation With Mannequin-Based Simulation for Improving Clinical Performances in Assessing and Managing Clinical Deterioration: Randomized Controlled Trial

**DOI:** 10.2196/jmir.3322

**Published:** 2014-09-17

**Authors:** Sok Ying Liaw, Sally Wai-Chi Chan, Fun-Gee Chen, Shing Chuan Hooi, Chiang Siau

**Affiliations:** ^1^Alice Lee Centre for Nursing StudiesYong Loo Lin School of MedicineNational University of SingaporeSingapore 117597Singapore; ^2^School of Nursing and MidwiferyFaculty of Health and MedicineThe University of NewcastleNewcastleAustralia; ^3^Department of AnaesthesiaYong Loo Lin School of MedicineNational University of SingaporeSingaporeSingapore; ^4^Department of PhysiologyYong Loo Lin School of MedicineNational University of SingaporeSingaporeSingapore

**Keywords:** simulation, education, virtual patient, deterioration, clinical performance, patient safety

## Abstract

**Background:**

Virtual patient simulation has grown substantially in health care education. A virtual patient simulation was developed as a refresher training course to reinforce nursing clinical performance in assessing and managing deteriorating patients.

**Objective:**

The objective of this study was to describe the development of the virtual patient simulation and evaluate its efficacy, by comparing with a conventional mannequin-based simulation, for improving the nursing students’ performances in assessing and managing patients with clinical deterioration.

**Methods:**

A randomized controlled study was conducted with 57 third-year nursing students who were recruited through email. After a baseline evaluation of all participants’ clinical performance in a simulated environment, the experimental group received a 2-hour fully automated virtual patient simulation while the control group received 2-hour facilitator-led mannequin-based simulation training. All participants were then re-tested one day (first posttest) and 2.5 months (second posttest) after the intervention. The participants from the experimental group completed a survey to evaluate their learning experiences with the newly developed virtual patient simulation.

**Results:**

Compared to their baseline scores, both experimental and control groups demonstrated significant improvements (*P*<.001) in first and second post-test scores. While the experimental group had significantly lower (*P*<.05) second post-test scores compared with the first post-test scores, no significant difference (*P*=.94) was found between these two scores for the control group. The scores between groups did not differ significantly over time (*P*=.17). The virtual patient simulation was rated positively.

**Conclusions:**

A virtual patient simulation for a refreshing training course on assessing and managing clinical deterioration was developed. Although the randomized controlled study did not show that the virtual patient simulation was superior to mannequin-based simulation, both simulations have demonstrated to be effective refresher learning strategies for improving nursing students’ clinical performance. Given the greater resource requirements of mannequin-based simulation, the virtual patient simulation provides a more promising alternative learning strategy to mitigate the decay of clinical performance over time.

## Introduction

The nurses’ role in recognizing, responding to, and reporting a patient’s clinical deterioration is critical in the prevention of the patient’s progression to cardiopulmonary arrest. A literature review identified an educational need to prepare nursing students to manage physiological deterioration of patients before they start their ward nursing practice [1]. A 6-hour mannequin-based simulation program, known as RAPIDS (Rescuing a Patient in Deteriorating Situations) was implemented as part of a core learning activity in an undergraduate nursing program. A randomized controlled study has shown that the RAPIDS program using ABDCDE (Airway, Breathing, Circulation, Disability, and Expose/Examine) and SBAR (Situation, Background, Assessment, and Recommendation) mnemonics effectively developed nursing students’ clinical competencies in assessing, managing deterioration, and communicating patient’s deterioration to the team doctor [2]. However, it is unclear how best to maintain these competencies post-RAPIDS training.

A qualitative study was conducted to examine the effect of the RAPIDS program on the nursing students’ performances in the clinical practice. The study showed the benefits of mannequin-based simulation in preparing the nursing students for their encounters with deteriorating ward patients. To optimize their retention and transfer of learning in RAPIDS, the study recommended regular reinforcement with follow-up simulation training using the ABCDE and SBAR mnemonics [3]. Previous studies have supported the use of mannequin-based simulation in the acquisition of clinical skills but have also demonstrated their limitations [1,4]. Because mannequin-based simulation involves a small number of students at one time, a considerable amount of faculty time is required to conduct repeated sessions. Besides faculty time, the availability of simulation facilities and scheduling issues are major challenges faced by educators when implementing mannequin-based simulation. These challenges made it difficult to be certain whether it is the best follow-up learning method to maintain or enhance previously acquired skills.

Virtual patient simulation has fewer of these resource constraints compared to mannequin-based simulation. It is capable of creating high-fidelity simulation by applying the features identified in a systematic review. With the capacity for exhibiting a high level of interactivity and realism, a wide range of clinical scenarios with guided reflection can be designed into the virtual patient simulation [5]. In addition, it can cater to a large number of learners simultaneously and be used by learners repeatedly when needed. Being accessible anytime and anywhere, it can also be integrated into curricula in a more flexible manner [6]. Although the use of virtual patient simulations have been widely adopted for training health professionals [7-9], more research is required to inform how to effectively design and integrate them into curricula [10,11].

A virtual patient simulation was designed and developed for use as an instructional learning strategy to revise RAPIDS training. We conducted a randomized controlled study to determine the efficacy of virtual patient simulation, by comparing it with mannequin-based simulation, in improving the nursing students’ clinical performances in assessing and managing deterioration. A survey was also conducted to evaluate learners’ perception towards the newly developed instructional strategy.

## Methods

### Design and Development of Virtual Patient Simulation

**Figure 1:**
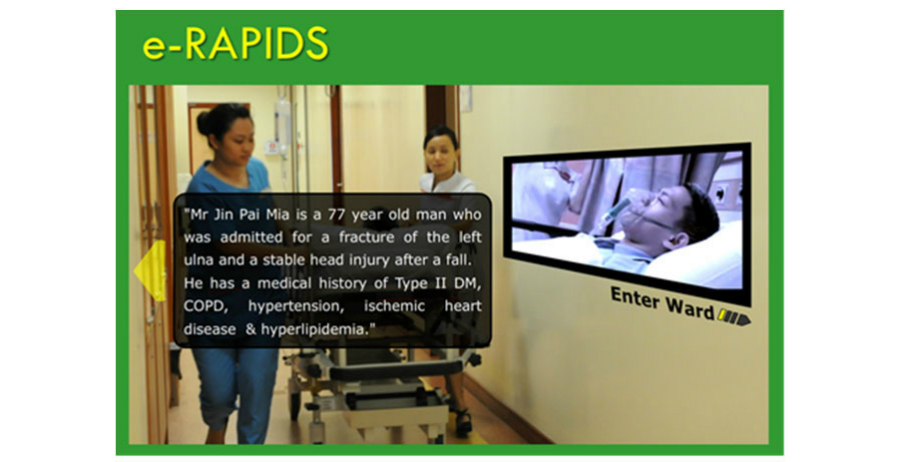
Clinical history of the virtual patient.

The virtual patient simulation, known as e-RAPIDS, was developed at National University of Singapore (NUS) by a group of academic staff, clinicians, and educational technologists. This single user interactive multimedia simulation was created using Flash software and run on a secure server hosted by NUS. The contents were developed based on the following learning objectives: (1) Demonstrate a systematic approach using the ABCDE mnemonic to assess and manage clinically deteriorated patient, and (2) Demonstrate effective communication skills using the SBAR tool to report patient deterioration to doctor. Five simulation scenarios associated with acute medical conditions (acute coronary syndrome, hypoglycemia, hypovolemic shock, sepsis, septic shock) were used. Common deteriorating conditions such as airway obstruction, breathlessness, hypotension, tachycardia, oliguria, altered consciousness, and abnormal temperature were embedded in these scenarios. All the scenarios applied the same clinical case history of a virtual patient who was admitted to a hospital with multiple medical conditions and comorbidities (Figure 1). The complexity of the case history allowed sequential simulation of deteriorating events at different phases of the virtual patient’s hospitalization. Appropriate clinical findings and responses of the virtual patient were developed for each scenario.

**Figure 2:**
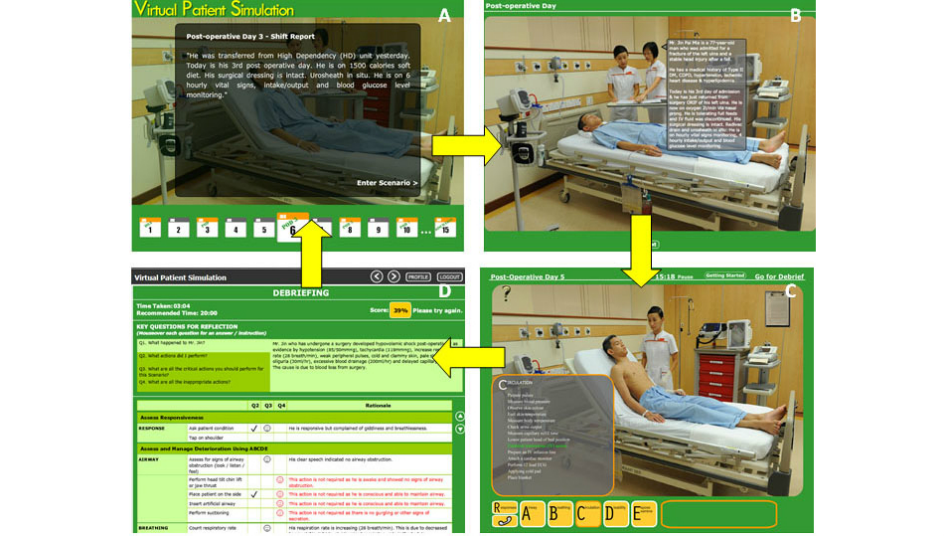
User follows A to D to navigate each scenario: A, Click on the patient’s day of admission to enter a scenario; B, Receive patient information during hand-off report; C, Emulate the role of nurse to assess and manage deteriorating patient by clicking on the ABCDE options menus; D, Self-reflection through a list of questions.

Figure 2 presents the path of virtual patient simulation scenarios. The learner can choose to participate in any scenarios by clicking on the patient’s day of admission (part A in Figure 2). The deteriorating events occur on first, third, sixth, eighth, and tenth day of admission. Once inside the virtual ward, the learner receives a handover report on the case history and the latest update of the virtual patient’s condition (part B in Figure 2). After the handover, the learner, who plays the role of the nurse, is presented with a virtual patient with deteriorating conditions. The learner is required to assess and manage the virtual patient’s deteriorating condition by clicking on the actions from the ABCDE control menus (part C in Figure 2). There are over 30 actions programmed into the simulation. The clicking of a specific action may lead to an arrow sign that directs the learner to click on specific equipment or an item in the virtual ward. Immediate feedback, including information and physiological changes, was programmed into the system to respond to the learner’s actions. The information generated from an action is delivered through the virtual nurse’s verbalization with texts displayed in the form of speech bubbles. The physiological parameters including heart rate, blood pressure, and oxygenation are displayed on an electronic monitor in the virtual ward. SBAR control menus are used in the program to aid the learner in reporting about the patient’s deterioration.

At the end of each scenario, the learner enters a “debriefing” screen (part D in Figure 2), which consists of (1) five debriefing questions, (2) an evaluation tool adapted from a validated and reliable tool known as RAPIDS tool, and (3) a performance score. The debriefing questions lead the learner to reflect on the deteriorating situation and actions they have taken. Using a checklist format and brief explanation, the evaluation tool provides feedback to the learners on the appropriate and inappropriate actions taken in the simulation scenario. A score is automatically calculated from the evaluation tool based on the learner’s actions in the scenario (part D in Figure 2).

### Evaluation of Virtual Patient Simulation

#### Design and Sample

**Figure 3:**
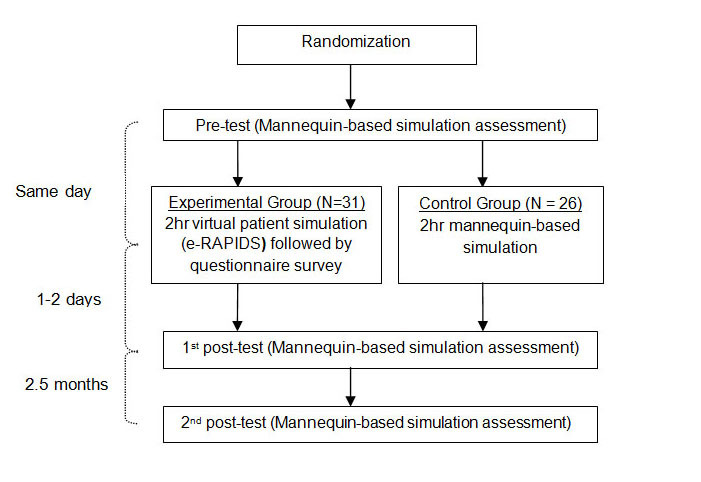
Flow of data collection.

A prospective, randomized controlled trial with a pretest-posttest design was conducted (see Figure 3). The study was approved by the National University of Singapore (NUS) Institutional Human Research Ethics Board. A total of 97 senior nursing students in their third year of nursing studies, who had undertaken a 6-hour mannequin-based RAPIDS simulation program about 8 months prior, from August to December 2011 at NUS, were invited to participate in the study through email. After being given a participant information sheet that explained the purpose of the study, 61 students consented to participate. The students were assured that their decision to participant or not to participate would not affect their training. They were randomly assigned to experiment (n=31) and control group (n=30) using a random number table. However, 4 participants from the control group withdrew from the study as they were unable to join the scheduled simulation, leaving that group with 26 students.

#### Baseline Testing

After randomization, both groups underwent baseline testing using a mannequin-based simulation assessment at the university simulation center. The participants from both groups undertook the tests individually. To reduce bias, the participants were required to wear caps, gowns, and masks to blind their identities from the raters who may have known the participants’ training background. Following an orientation to the simulated ward and brief introduction of the case history, a mannequin-based test scenario with signs and symptoms of clinical deterioration was presented to the participants. The participants were each given 15 minutes to assess and manage the deteriorating patient simulator with signs of shock. As all the participants undertook the same test scenario, they were reminded about the confidentiality of the scenario before they left the laboratory. The entire process of the testing scenario was videotaped.

#### Intervention

The interventions for both groups were conducted immediately after the baseline testing. Both interventions were conducted concurrently on the same day in the university’s simulation center. The duration of intervention (2 hours) was equal in the experimental and control groups. The participants in the experimental group were brought individually into a room with a computer set up to use the virtual patient simulation as described above. They were instructed to undertake all the simulation scenarios in the virtual patient simulation. The participants from the control group were placed into groups of 6 to participate in the mannequin-based simulation, led by a trained simulation facilitator. The participants worked through two simulation scenario (sepsis and septic shock) of a patient with deteriorating conditions. These scenarios were developed to model situations that led the learners to use ABCDE and SBAR mnemonics to perform thorough nursing assessment and management, and to call for help. Each simulation scenario began with a role play followed by a debriefing session. While 3 participants undertook the role play, the rest of the learners observed the scene. To allow all participants to have hands-on experience, the participants reversed their roles as observers and role players in the two scenarios. During debriefing, the facilitator used questioning techniques to lead the learners to reflect their simulation performances. In addition, using the ABCDE and SBAR mnemonic checklists, the facilitator provided specific feedback to the learners.

#### Survey and Posttests

On completion of the virtual patient simulation, the participants in the experimental group were asked to complete a questionnaire to evaluate their learning experiences. All participants from both groups undertook two posttests using mannequin-based simulation assessment. The first posttest was conducted one or two days after the intervention. This was followed by the second posttest about 2.5 months later. The scenario and instrument used for the posttest simulation assessment were similar to the baseline testing. The entire process of the simulation assessment was videorecorded.

#### Instruments

The recorded simulation performances were observed and rated by an academic staff using the RAPIDS tool, which consists of two domains: (1) ABCDE domain consists of binary checklist items and a global rating scale item to evaluate the performance in assessing and managing patient deterioration, and (2) SBAR domain consists of binary checklist items and a global rating scale to measure the communications skills in reporting patient deterioration. The psychometric properties of the RAPIDS tool, including content and construct validity, and interrater reliability were tested and supported in a previous study [12]. An excellent interrater reliability between 2 raters, with high intraclass correlation coefficient of .92 (95% CI 0.82-0.96), was obtained in this study.

A 19-item questionnaire with four subscales (system quality, information quality, user satisfaction, and net benefit) was adapted and modified from the e-learning systems success (ELSS) scale to evaluate the participants’ perception of the virtual patient simulation. Each item is rated on a 7-point Likert-type scale ranging from “strongly disagree” to “strongly agree”. The scale was developed and tested by Wang et al [13] in a previous study to measure the success of an e-learning system in an organizational context. A high internal consistency of this scale was obtained in this study (Cronbach alpha=.904).

#### Data Analysis

The sample size calculation was based on a previous study measured using the RAPIDS tool. A sample of 15 participants per arm gave an effect size of 3.16 that could achieve more than 80% power at 5% alpha level [14]. Allowing for an attrition rate of 50%, particularly from the second posttest, the aim was to recruit 30 participants to each arm. Chi-square tests and *t* tests were used to examine any differences in demographic characteristics between the two groups. Interrater reliability was assessed by intraclass correlation coefficient. Repeated measures analysis of variance (ANOVA) was used to analyze time and group differences in the performance scores. Descriptive statistics using means and standard deviation were performed to examine the participants’ perception of the virtual simulation.

## Results

Most of the participants were female (89.5%, 51/57), Chinese (78.9%, 45/57), and an average of 21.86 years old (SD 1.13). The intervention and control group did not differ significantly in demographic variables of gender (*P*=.29), ethnic (*P*=.07), and age (*P*=.14). These results supported the randomization and homogeneity of the participants between the groups.

**Figure 4:**
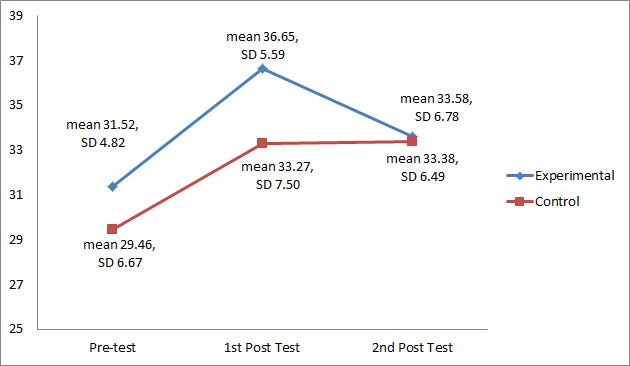
Performance mean scores and standard deviation at pretest, first posttest, and second posttest.

With the possible maximum performance mean scores of 54, the score of 30.58 (SD 5.78) indicated that most participants had an average performance score. Repeated-measures ANOVA (within-subject analysis) indicated a significant increase in the first posttest scores from the pretest scores for the experimental (*P*<.001) and control groups (*P*<.05). As shown in Figure 4, the second posttest scores for the experimental group decreased significantly (*P*<.05) from the first posttest. However, no significant difference (*P*=.94) between the first and second posttest scores was found for the control group. The second posttest scores for the experimental (*P*<.05) and control groups (*P*<.01) were significantly higher from the pretest scores.

Pairwise comparison between the two groups at the three time-points indicated that both groups increased significantly from pretest to first posttest (*P*<.001) and from the pretest to second posttest (*P*<.001). There was no significant difference between the first and second posttests for both groups (*P*=.12). Repeated-measures ANOVA (between-subject difference) showed that there was no significant difference in performance mean scores over time between the experimental and control group (*P*=.17).

The mean scores from the participants’ rating (experimental group) on a 7-point scale indicated that that they were satisfied with the virtual patient simulation (mean 6.06, SD 0.71), highly positive about the quality of the system (mean 6.01, SD 0.56) and information (mean 6.06, SD 0.50), and perceived highly the net benefits (mean 6.28, SD 0.59) of the program.

## Discussion

### Principal Findings

The virtual patient simulation for this study was developed through a systematic and comprehensive evaluation of a mannequin-based simulation program known as RAPIDS program. Programmatic research was conducted previously from the beginning, from performing needs analysis to evaluating the long-term outcomes of the RAPIDS program in clinical practice [1-3]. The scenarios were developed based on common acute care events and opinions of clinical experts. A variety of conceptual frameworks were applied in developing and implementing the virtual patient simulation. The ABCDE and SBAR mnemonics, accepted guidelines for the care of critically ill patients, form the foundation for the training contents. Kolb’s experiential learning guided the design and process of the virtual patient simulation in which the learners began the learning process in each scenario with a simulation experience followed by self-reflection [2].

The effectiveness of the virtual patient simulation was evaluated by comparing it with mannequin-based simulation. Comparing between these two simulations modalities, we acknowledged that the variations in structures and designs were recognized as confounding variables. It is important to address the confounding variables for the observed results [15]. Using a self-directed learning approach, the virtual patient simulation provided learners control of their training agenda, allowing repeated “deliberate practice” and receiving standardized feedback. The mannequin-based simulation used a collaborative learning approach that required the learners to perform hands-on simulation in small groups and engaged in a group debrief led by a trained facilitator. With this confounding variable, it is crucial to provide evidence to support the validity of the outcome measure [15]. The outcome measure selected for this study was closely aligned with the learning objectives. In both the virtual patient simulation and mannequin-based simulation, the ABCDE and SBAR mnemonics provided the frameworks to guide learning of the training contents. They were also constructs for the RAPIDS evaluation tool, which was previously validated and used to measure the performance outcomes during the simulation-based assessment [1]. The validity of the RAPIDS tool has been well established in a previous study, based on consensus among a panel of clinical experts [13].

The performance scores (baseline) from the simulation-based assessment, conducted 8 months following the full-scale RAPIDS simulation course, indicated that there is much room for the participants to improve their performance in assessing and managing patient deterioration. We found that both virtual patient simulation and mannequin-based simulation are effective refresher learning strategies to improve the nursing students’ clinical performance. Earlier studies did not support the use of virtual patient as part of a blended approach to advanced cardiac life support training (ACLS) [16,17]. It was suggested that virtual patient simulation may not be the best modality for learning ACLS, which is based on algorithmic approaches and psychomotor skill development, but better used to develop decision making skills [18]. In this study, we designed virtual patient simulation to promote clinical reasoning skills through deliberate practice with multiple and varied simulation scenarios. Despite the significant improvement of the posttest scores in both the experimental and controls groups, the brief refresher training had generally not achieved an optimal level of skill improvement. The learning from the virtual patient simulation could be maximized by allowing the learners to undertake the learning on a regular basis. The positive evaluation of the virtual patient simulation by the nursing students supported the use of this learning strategy in the nursing undergraduate curriculum in our institution.

We found that both virtual patient and mannequin-based simulation were effective in improving nursing students’ ability to assess and manage a deteriorating patient. This finding is consistent with a previous study that demonstrated the effectiveness of both virtual simulation and mannequin-based simulation for improving teamwork skills [4]. We incorporated the features for effective learning identified by a systematic review into our simulation design [19]. These principles included curriculum integration, range of training levels, clinical variation, multiple learning strategies, deliberate practice, and feedback. The virtual patient simulation was integrated into a final year nursing module within the existing undergraduate curricula. It was used as a self-directed learning strategy to maintain competence following a RAPIDS course. A variety of simulation scenarios was developed for virtual patient simulation to provide clinical variation. These scenarios were sequenced across a range of training levels, progressively from simple to complex problem solving, to facilitate deliberate practice. In each scenario, the learners engaged in active learning by playing the role of a nurse in assessing and managing the deteriorating virtual patient. The learners received direct real-time feedback from the computer software with pre-programmed patient responses based on their nursing actions. At the end of each scenario, the learner also received feedback via the scoring system and checklist that appears in the debriefing screen. As our study provided positive learning outcomes of the virtual patient simulation, we call upon educators to apply the principles of simulation in the design and implementation of virtual patient learning experiences.

Although the use of virtual patient simulation yielded immediate improvement in the participants’ clinical performance, this improvement was not sustained at 2.5 months. The participants in the mannequin-based simulation, however, demonstrated a more consistent and sustained improvement at 2.5 months, with little decay over time. This retention of learning suggests that hands-on simulation provided deeper learning compared to multimedia teaching modalities, which is consistent with the result of a previous study [20]. In our study, the virtual patient simulation exposed the participants to a larger number of cases with repetitive practice and provided a significant amount of didactic information in the feedback section. These aspects were much less integrated into the mannequin-based simulation, which also relied on collaborative learning and facilitator-led debriefing. The social interaction underlying collaborative learning has been found to promote long-term retention of the learned material [21]. In addition, learning gets deepened with guided and thoughtful reflection rather than mere simulation experiences and customized feedback [22].

As both learning tools are associated with improved outcome, comparing their resource intensity may lead to better-informed curricular decisions [23]. The virtual patient simulation was a more resource-efficient simulation modality than the mannequin-based simulator for a refresher training course. Although there were initial start-up costs for developing the virtual patient simulation, its implementation was less resource intensive than the mannequin-based simulation. The use of virtual patient simulation does not require simulation facilitators, expensive equipment, or facilities, which are associated with high costs. In addition, it can accommodate multiple users at one time and provide learning content for a large group of learners, all of which can facilitate efficiencies in curriculum planning and use of instructor time [24]. As a result, the use of virtual patient simulation would be a viable option in institutions with large numbers of learners [25]. While it is also feasible to repeat the mannequin-based simulation to maintain competence, it has a higher resource demand. The opportunities to engage in repetitive training in the virtual patient simulation are unlimited. This repetitive learning is essential if the learner is to achieve long-term retention of learning [26].

### Limitations

There are limitations that warrant attention. First, when comparing the virtual patient simulation with the mannequin-based simulation, it was challenging to account for all the differences in the simulation designs and structure. As such, the comparison was confounded [27], and the findings may not be reliably generalized. Second, we did not measure the level of performance immediately after the 6-hour RAPIDS simulation course to determine the level of deterioration prior to the refresher training. Third, in the present study, both groups were given the same duration of training. We did not optimize the use of the virtual patient simulation by allowing the experimental group to undertake the learning on a regular basis. Future study is needed to find out whether frequent exposure of virtual patient simulation could prevent the decay of learning. Fourth, due to faculty and students’ time constraints, the outcome measure was limited to immediate and 2.5 months following the intervention. Future study could evaluate the competence over a longer period of time. Fifth, we did not evaluate the learning experience from the control group. A comparative study on learners’ experiences with the two types of simulation modalities could be considered for future study. Last, the outcome measure was limited to individual simulation-based assessment, which may bias towards the experimental group. Future studies could determine the outcomes of the virtual patient simulation on actual clinical practice.

### Conclusions

A virtual patient simulation, known as e-RAPIDS, was developed as a refresher training course to enhance nursing students’ clinical performance in rescuing a patient in a deteriorating situation. Although the study did not show the superiority of virtual simulation over mannequin-based simulation, both simulations have shown to be effective learning strategies for improving clinical performance in assessing and managing clinical deterioration. Overall, the learners evaluated the virtual patient simulation very positively. Given the flexibility, practicality, and scalability of the virtual patient simulation, it appears to provide a more promising learning strategy over time than the mannequin-based simulation for refreshing clinical performance. Further studies can build on this to provide more evidence on blended learning, where the virtual simulation is integrated with the mannequin-based simulation.

## Multimedia Appendix 1

CONSORT-EHEALTH checklist V1.6.2 [28].

## Abbreviations

ABDCDE: airway, breathing, circulation, disability, and expose/examine
ACLS: advanced cardiac life support
ANOVA: analysis of variance
NUS: National University of Singapore
RAPIDS: rescuing a patient in deteriorating situations
SBAR: situation, background, assessment, and recommendation
