# Supplementary material for: Comparison of Virtual Patient Simulation With Mannequin-Based Simulation for Improving Clinical Performances in Assessing and Managing Clinical Deterioration: Randomized Controlled Trial
Source: J Med Internet Res. 2014 Sep 17;16(9):e214. doi: 10.2196/jmir.3322 (PMC4180357; doi:10.2196/jmir.3322)
Supplement: Supplementary file 1 [file jmir_v16i9e214_app1.pdf]

CONSORT-EHEALTH Checklist V1.6.2 Report

(based on CONSORT-EHEALTH V1.6), available at [http://tinyurl.com/consort-ehealth-v1-6].

3322

Date completed

2/11/2014 10:22:33

by

Sok Ying Ilaw

Virtual Patient is as Effective as Mannequin-based Simulator for Improving Performances in Assessing and Managing Clinical Deterioration: Randomized Controlled Trial

TITLE

1a-i) Identify the mode of delivery in the title

"Virtual Patient is as Effective as Mannequin-based Simulator"

1a-ii) Non-web-based components or important co-interventions in title

"Virtual Patient is as Effective as Mannequin-based Simulator for Improving Performances in Assessing and Managing Clinical Deterioration"

1a-iii) Primary condition or target group in the title

"Improving Performances in Assessing and Managing Clinical Deterioration"

ABSTRACT

1b-i) Key features/functionalities/components of the intervention and comparator in the METHODS section of the ABSTRACT

"This study is to describe the development of the virtual patient simulation and evaluating its effectiveness, by comparing with mannequin-based simulation, for improving the clinical performance of nursing students in assessing and managing patients with clinical deterioration."

1b-ii) Level of human involvement in the METHODS section of the ABSTRACT

"the experimental group received 2-hour fully automated virtual patient simulation while the control group received 2-hour facilitator-led mannequin-based simulation training."

1b-iii) Open vs. closed, web-based (self-assessment) vs. face-to-face assessments in the METHODS section of the ABSTRACT

"A randomized controlled study was conducted with 57 third year nursing students who were recruited through email. After a baseline evaluation of all participants' clinical performance in a simulated environment, the experimental group received 2-hour fully automated virtual patient simulation while the control group received 2-hour facilitator-led mannequin-based simulation training. All participants were then re-tested one day (immediate post-test) and 2.5 months (retention post-test) after the intervention. The participants from the experimental group completed a survey to evaluate their learning experiences with virtual patient simulation."

1b-iv) RESULTS section in abstract must contain use data

"A randomized controlled study was conducted with 57 third year nursing students who were recruited through email."

1b-v) CONCLUSIONS/DISCUSSION in abstract for negative trials

Not applicable as trial was not negative

INTRODUCTION

2a-i) Problem and the type of system/solution

"To optimize their retention and transfer of learning in RAPIDS, the study recommended regular reinforcement with follow-up simulation training using the ABCDE and SBAR mnemonics [3]. Previous studies have supported the use of mannequin-based simulation in the acquisition of clinical skills, but have also demonstrated their limitations [1, 4]. As mannequin-based simulation involved a small number of students at one time, a considerable amount of faculty time is required to conduct repeated sessions. Besides faculty time, the availability of simulation facilities and timetabling issues are major challenges faced by educators when implementing mannequin-based simulation. These challenges made it difficult to be certain whether it is the best follow-up learning method to maintain or enhance previous acquired skills."

2a-ii) Scientific background, rationale: What is known about the (type of) system

"Computer-based simulation has fewer of these resources constraints compared to mannequin-based simulation. It can cater to a large number of learners simultaneously and be used by learners repeatedly when needed. Being accessible at anytime and anywhere, it can also be integrated into curricula in a more flexible manner [5]. Interactive computer simulations of clinical scenarios featuring virtual patients, have been widely adopted for training health professionals [6,7, 8]. The virtual patient is capable for creating high-fidelity simulation by applying the features identified in a systematic review. With the capacity for exhibiting a high level of interactivity and realism, a wide range of clinical scenarios with guided reflection can be designed into the virtual simulation technology [9]. However, more research is required to inform how to effectively design and integrate virtual patients into curricula [10,11]."

METHODS

3a) CONSORT: Description of trial design (such as parallel, factorial) including allocation ratio

"We conducted a randomized controlled study to determine the efficacy of e-RAPIDS training, by comparing with mannequin-based simulation, in improving the nursing students' clinical performances in assessing and managing deterioration. A survey was also conducted to evaluate the learners' perception towards the newly developed instructional strategy."

3b) CONSORT: Important changes to methods after trial commencement (such as eligibility criteria), with reasons

No changes after trial commencement

3b-i) Bug fixes, Downtimes, Content Changes

No bug fixes or unexpected events

4a) CONSORT: Eligibility criteria for participants

"Senior nursing students in their third year of nursing studies, who had undertaken a 6-hours mannequin-based RAPIDS simulation program from the period of August to December 2011 at NUS, were invited to participate in the study through email."

4a-i) Computer / Internet literacy

Not applicable as study samples are university students who are expected to be computer / internet literate.

4a-ii) Open vs. closed, web-based vs. face-to-face assessments:

"Senior nursing students in their third year of nursing studies, who had undertaken a 6-hours mannequin-based RAPIDS simulation program from the period of August to December 2011 at NUS, were invited to participate in the study through email."

4a-iii) Information giving during recruitment

"61 students consented to participate in the research study after they were given a participant information sheet that explained the purpose of the study."

4b) CONSORT: Settings and locations where the data were collected

"The experimental group then received a 2-hour e-RAPIDS whilst the control group received a 2-hour mannequin-based simulation. Both interventions were conducted concurrently on the same day in the University's simulation centre."

4b-i) Report if outcomes were (self-)assessed through online questionnaires
Not applicable as no online self-assessment was used
4b-ii) Report how institutional affiliations are displayed
Not applicable as all potential participants are recruited from the same hospital
5) CONSORT: Describe the interventions for each group with sufficient details to allow replication, including how and when they were actually administered
5-i) Mention names, credential, affiliations of the developers, sponsors, and owners
"The e-RAPIDS was developed at National University of Singapore (NUS) by a group of academic staff, clinicians and educational technologists. This interactive multimedia simulation was created using Flash software and run on a secure server hosted by NUS."
5-ii) Describe the history/development process
Not applicable as this is a newly developed software
5-iii) Revisions and updating
Not applicable as this is a newly developed software
5-iv) Quality assurance methods
"The e-RAPIDS was developed at National University of Singapore (NUS) by a group of academic staff, clinicians and educational technologists."
5-v) Ensure replicability by publishing the source code, and/or providing screenshots/screen-capture video, and/or providing flowcharts of the algorithms used
"The e-RAPIDS was designed based on Kolb's experiential learning. Five simulation scenarios associated with acute medical conditions (acute coronary syndrome, hypoglycaemia, hypovolemic shock, sepsis, septic shock) were used. It focuses on rescuing a patient in deteriorating situation with the following learning objectives: (1) Demonstrate a systematic approach using the ABCDE mnemonic to assess and manage clinically deteriorated patient; (2) Demonstrate effective communication skills using the SBAR tool to report patient deterioration to doctor.
Common deteriorating conditions such as airway obstruction, breathlessness, hypotension, tachycardia, oliguria, altered consciousness and abnormal temperature were embedded in these scenarios. All the scenarios applied the same clinical case history of a virtual patient who was admitted to a hospital with multiple medical conditions and co-morbidities (Figure 1). The complexity of the case history allowed sequential simulation of deteriorating events at different phases of the virtual patient's hospitalization. Appropriate clinical findings and responses of the virtual patient were developed for each scenario. Upon entering the virtual ward, the user can choose to participate in the different scenarios by clicking on the patient's day of admission. Each scenario begins with a handover report to acquaint the learners with the case history. After the report, the user, who plays the role of the nurse, is presented with a virtual patient with deteriorating conditions (Figure 2). The user is required to assess and manage the virtual patient's deteriorating condition by selection of actions from the ABCDE control menus. Immediate feedback, including physiological changes, was programmed into the system to respond to the learner's actions. SBAR control menus are used in the program to aid the learner to report about the patient's deterioration. At the end of each scenario, the user enters a "debriefing" screen (Figure 3), which consists of: (1) five debriefing questions; (2) an evaluation tool adapted from a validated and reliable tool known as RAPIDS-tool, and (3) a performance score. The debriefing questions lead the learner to reflect on the deteriorating situation and actions they have taken. Using a checklist format and brief explanation, the evaluation tool provides feedback to the learners on the appropriate and inappropriate actions taken in the simulation scenario. A score is automatically calculated from the evaluation tool based on the user's actions in the scenario."
5-vi) Digital preservation
Screenshots of the program were provided as Figures
5-vii) Access
"The participants in the experimental group were brought individually into a room with computer setup to use the e-RAPIDS."
5-viii) Mode of delivery, features/functionalities/components of the intervention and comparator, and the theoretical framework
"The e-RAPIDS was designed based on Kolb's experiential learning. Five simulation scenarios associated with acute medical conditions (acute coronary syndrome, hypoglycaemia, hypovolemic shock, sepsis, septic shock) were used. It focuses on rescuing a patient in deteriorating situation with the following learning objectives: (1) Demonstrate a systematic approach using the ABCDE mnemonic to assess and manage clinically deteriorated patient; (2) Demonstrate effective communication skills using the SBAR tool to report patient deterioration to doctor.
Common deteriorating conditions such as airway obstruction, breathlessness, hypotension, tachycardia, oliguria, altered consciousness and abnormal temperature were embedded in these scenarios. All the scenarios applied the same clinical case history of a virtual patient who was admitted to a hospital with multiple medical conditions and co-morbidities (Figure 1). The complexity of the case history allowed sequential simulation of deteriorating events at different phases of the virtual patient's hospitalization. Appropriate clinical findings and responses of the virtual patient were developed for each scenario. Upon entering the virtual ward, the user can choose to participate in the different scenarios by clicking on the patient's day of admission. Each scenario begins with a handover report to acquaint the learners with the case history. After the report, the user, who plays the role of the nurse, is presented with a virtual patient with deteriorating conditions (Figure 2). The user is required to assess and manage the virtual patient's deteriorating condition by selection of actions from the ABCDE control menus. Immediate feedback, including physiological changes, was programmed into the system to respond to the learner's actions. SBAR control menus are used in the program to aid the learner to report about the patient's deterioration. At the end of each scenario, the user enters a "debriefing" screen (Figure 3), which consists of: (1) five debriefing questions; (2) an evaluation tool adapted from a validated and reliable tool known as RAPIDS-tool, and (3) a performance score. The debriefing questions lead the learner to reflect on the deteriorating situation and actions they have taken. Using a checklist format and brief explanation, the evaluation tool provides feedback to the learners on the appropriate and inappropriate actions taken in the simulation scenario. A score is automatically calculated from the evaluation tool based on the user's actions in the scenario."
5-ix) Describe use parameters
"The experimental group then received a 2-hour e-RAPIDS whilst the control group received a 2-hour mannequin-based simulation."
5-x) Clarify the level of human involvement
"The participants in the experimental group were brought individually into a room with computer setup to use the e-RAPIDS."........The participants from the control group were placed into groups of 6 to participate in the mannequin-based simulation, led by a trained simulation facilitator."
5-xi) Report any prompts/reminders used
Not applicable as no prompts or reminders were used.
5-xii) Describe any co-interventions (incl. training/support)
Not applicable as the virtual simulation is a standalone intervention.
6a) CONSORT: Completely defined pre-specified primary and secondary outcome measures, including how and when they were assessed

"The pre-test and post-tests on simulation-based assessment took place at the University simulation centre. The participants from both groups undertook the tests individually. The participants were required to wear caps, gowns and masks to blind their identities from the raters. Following an orientation to the simulated ward and brief introduction of the case history, a mannequin-based test scenario with signs and symptoms of clinical deterioration was presented to the participants. The participants were each given 15 minutes to assess and manage the deteriorating patient simulator with signs of shock. As all the participants undertook the same test scenario, they were reminded about the confidentiality of the scenario before they left the laboratory. The entire process of the testing scenario was videotaped. The recorded performances were observed and rated by an academic staff using the RAPIDS-tool. ABCDE domain consists of binary checklist item and a global rating scale item to evaluate the performance in assessing and managing patient deterioration; (2) SBAR domain comprises of binary checklist items and a global rate scale to measure the communications skills in reporting patient deterioration. The psychometrics properties of the RAPIDS- tool, including content and construct validity, and inter-rater reliability were tested and supported in a previous study [12]. An excellent inter-rater reliability between two raters, with high intraclass correlation coefficient (ICC) of 0.92 (95% confident interval, 0.82-0.96), was obtained in this study. A 19-item with four subscales (system quality, information quality, user satisfaction and net benefit) was adapted and modified from the e-learning systems success (ELSS) scale to explore the participants' perception of the e-RAPIDS. The scale was developed and tested by Wang et al [13] in a previous study. A high internal consistency of this scale with Cronbach's alpha 0.904 was obtained in this study."

6a-i) Online questionnaires: describe if they were validated for online use and apply CHERRIES items to describe how the questionnaires were designed/deployed

Not applicable as no online questionnaires was used.

6a-ii) Describe whether and how "use" (including intensity of use/dosage) was defined/measured/monitored

Not applicable

6a-iii) Describe whether, how, and when qualitative feedback from participants was obtained

"The pre-test and post-tests on simulation-based assessment took place at the University simulation centre. The participants from both groups undertook the tests individually. The participants were required to wear caps, gowns and masks to blind their identities from the raters. Following an orientation to the simulated ward and brief introduction of the case history, a mannequin-based test scenario with signs and symptoms of clinical deterioration was presented to the participants. The participants were each given 15 minutes to assess and manage the deteriorating patient simulator with signs of shock. As all the participants undertook the same test scenario, they were reminded about the confidentiality of the scenario before they left the laboratory. The entire process of the testing scenario was videotaped. The recorded performances were observed and rated by an academic staff using the RAPIDS-tool. ABCDE domain consists of binary checklist item and a global rating scale item to evaluate the performance in assessing and managing patient deterioration; (2) SBAR domain comprises of binary checklist items and a global rate scale to measure the communications skills in reporting patient deterioration. The psychometrics properties of the RAPIDS- tool, including content and construct validity, and inter-rater reliability were tested and supported in a previous study [12]. An excellent inter-rater reliability between two raters, with high intraclass correlation coefficient (ICC) of 0.92 (95% confident interval, 0.82-0.96), was obtained in this study. A 19-item with four subscales (system quality, information quality, user satisfaction and net benefit) was adapted and modified from the e-learning systems success (ELSS) scale to explore the participants' perception of the e-RAPIDS. The scale was developed and tested by Wang et al [13] in a previous study. A high internal consistency of this scale with Cronbach's alpha 0.904 was obtained in this study."

6b) CONSORT: Any changes to trial outcomes after the trial commenced, with reasons

Not applicable as no changes occurred after the trial commenced

7a) CONSORT: How sample size was determined

7a-i) Describe whether and how expected attrition was taken into account when calculating the sample size

The sample size calculation was based upon a previous study measured using the RAPIDS-tool. A sample of 15 participants per arm gave an effect size of 3.16 that could achieve more than 80% power at 5% alpha level [14]. Allowing for an attrition rate of 50%, particularly from the retention post-test, the aim was to recruit 30 participants to each arm.

7b) CONSORT: When applicable, explanation of any interim analyses and stopping guidelines

No applicable as no interim analysis was conducted and no stopping guidelines were defined.

8a) CONSORT: Method used to generate the random allocation sequence

"They were randomly assigned to experiment (N = 31) and control group (N = 30) using a random number table."

8b) CONSORT: Type of randomisation; details of any restriction (such as blocking and block size)

"61 students consented to participate in the research study after they were given a participant information sheet that explained the purpose of the study. They were randomly assigned to experiment (N = 31) and control group (N = 30) using a random number table."

9) CONSORT: Mechanism used to implement the random allocation sequence (such as sequentially numbered containers), describing any steps taken to conceal the sequence until interventions were assigned

"They were randomly assigned to experiment (N = 31) and control group (N = 30) using a random number table."

10) CONSORT: Who generated the random allocation sequence, who enrolled participants, and who assigned participants to interventions

"They were randomly assigned to experiment (N = 31) and control group (N = 30) using a random number table."

11a) CONSORT: Blinding - If done, who was blinded after assignment to interventions (for example, participants, care providers, those assessing outcomes) and how

11a-i) Specify who was blinded, and who wasn't

"The participants from both groups undertook the tests individually. The participants were required to wear caps, gowns and masks to blind their identities from the raters."

11a-ii) Discuss e.g., whether participants knew which intervention was the "intervention of interest" and which one was the "comparator"

Not applicable as 2 interventions were used and both were of interest.

11b) CONSORT: If relevant, description of the similarity of interventions

Not applicable as the comparator is not a placebo

12a) CONSORT: Statistical methods used to compare groups for primary and secondary outcomes

"Chi-square test and t-test were used to examine any differences in demographic characteristics between the two groups. Inter-rater reliability was assessed by intraclass correlation coefficient (ICC). Paired t-test was used to evaluate any change between baseline and post-test scores for each subscale and domain. ANCOVA was used to evaluate the effect of the intervention program on post-test scores by using the baseline scores as a covariate. Descriptive statistics using means and standard deviation were performed to examine the participants' satisfaction, self-confidence in learning and perception of the simulation features."

12a-i) Imputation techniques to deal with attrition / missing values

Not applicable as no missing values was reported.

12b) CONSORT: Methods for additional analyses, such as subgroup analyses and adjusted analyses

Not applicable as subgroup analyses was not applied

RESULTS

13a) CONSORT: For each group, the numbers of participants who were randomly assigned, received intended treatment, and were analysed for the primary outcome

"They were randomly assigned to experiment (N = 31) and control group (N = 30) using a random number table. However, four participants from the experimental group withdrew from the study as they were unable to join the scheduled simulation, leaving the group with 26 students."

13b) CONSORT: For each group, losses and exclusions after randomisation, together with reasons

"four participants from the experimental group withdrew from the study as they were unable to join the scheduled simulation, leaving the group with 26 students."

13b-i) Attrition diagram

14a) CONSORT: Dates defining the periods of recruitment and follow-up

"Senior nursing students in their third year of nursing studies, who had undertaken a 6-hours mannequin-based RAPIDS simulation program from the period of August to December 2011 at NUS, were invited to participate in the study through email."

14a-i) Indicate if critical "secular events" fell into the study period

14b) CONSORT: Why the trial ended or was stopped (early)

Ended after completion of the intervention

15) CONSORT: A table showing baseline demographic and clinical characteristics for each group

A table was included

15-i) Report demographics associated with digital divide issues

"Most of the participants were female (89.5%), Chinese (78.9%), with an average of 21.86 years old (SD = 1.13). The intervention and control group did not differ significantly in demographic variables of gender ($X2 = 1.15$, $p = 0.29$), ethnic ($X2 = 7.02$, $p = 0.07$) and age ($t = 1.52$, $p = 0.14$). These results supported the randomization and homogeneity of the participants between the groups. "

16a) CONSORT: For each group, number of participants (denominator) included in each analysis and whether the analysis was by original assigned groups

16-i) Report multiple "denominators" and provide definitions

Not applicable

16-ii) Primary analysis should be intent-to-treat

17a) CONSORT: For each primary and secondary outcome, results for each group, and the estimated effect size and its precision (such as 95% confidence interval)

"Most of the participants were female (89.5%), Chinese (78.9%), with an average of 21.86 years old (SD = 1.13). The intervention and control group did not differ significantly in demographic variables of gender ($X2 = 1.15$, $p = 0.29$), ethnic ($X2 = 7.02$, $p = 0.07$) and age ($t = 1.52$, $p = 0.14$). These results supported the randomization and homogeneity of the participants between the groups. With the possible maximum performance score of 54, the means score of 30.58 (SD 5.78) indicates that most participants have an average performance score. Figure 1 shows that at pre-test, the performance scores for experimental and control groups, did not differ significantly ($t =1.35$, $p =0.18$). Immediately after the intervention, the experimental ($t = 5.18$, $p < 0.001$) and control group ($t = 2.67$, $p < 0.05$), demonstrated significant increase in the immediate post-scores from the pre-test scores. Between-group comparison indicated no significant difference ($F = 3.83$, $p = 0.06$) in the immediate post test scores between the two groups after controlling the pre-test score.As shown in Figure 1, the retention post-test scores for the experimental group decreased significantly ($t = 2.53$, $p < 0.05$) from the immediate post-test. However, no significant difference ($t = 0.08$, $p = 0.49$) between the immediate and retention post-test scores was found for the control group. The retention post-test scores for the experimental ($t = 2.07$, $p < 0.05$) and control groups ($t = 3.11$, $p < 0.01$) were significantly higher from the pre-test scores. Between-group comparison indicated no significant difference ($F = 0.52$, $p = 0.47$) in the retention post-test scores between the two groups after controlling the pre-test scores. The means scores from the participants' rating (experimental group) on seven-point scale indicated that that they were satisfied with the e-RAPIDS (M=6.06, SD=0.71), highly positive with the quality of the system (M=6.01, SD=0.56) and information (M= 6.06, SD=0.50), and perceived highly the net benefits (M=6.28, SD=0.59) of the program."

17a-i) Presentation of process outcomes such as metrics of use and intensity of use

No process outcomes defined and measured

17b) CONSORT: For binary outcomes, presentation of both absolute and relative effect sizes is recommended

No binary outcomes were measured

18) CONSORT: Results of any other analyses performed, including subgroup analyses and adjusted analyses, distinguishing pre-specified from exploratory

Not applicable as not other analysis was performed

18-i) Subgroup analysis of comparing only users

Not applicable as no subgroup analysis was performed

19) CONSORT: All important harms or unintended effects in each group

No harms and intended effects occurrd

19-i) Include privacy breaches, technical problems

Not applicable as there is no occurence

19-ii) Include qualitative feedback from participants or observations from staff/researchers

No qualitative feedback measured

DISCUSSION

20) CONSORT: Trial limitations, addressing sources of potential bias, imprecision, multiplicity of analyses

20-i) Typical limitations in ehealth trials

"There are limitations that warrant attention. First, when comparing the e-RAPIDS with the mannequin-based simulation, it was challenging to account all the differences in the simulation designs and structure. As such the comparison was confounded [26], and the findings may not be reliably generalized. Second, we did not measure the level of performance immediately after the 6-hr RAPIDS simulation course to determine the level of deterioration prior to the refresher training. Third, in the present study, both groups were given the same duration of training. We did not optimize the use of the e-RAPIPS by allowing the experimental group to undertake the learning on a regular basis. Future study is needed to find out whether frequent exposure of e-RAPIDS could prevent the decay of learning. Fourth, due to faculty and students' time constraints, the outcome measure was limited to immediate and 2.5 months following the intervention. Future study could evaluate the competence over a longer period of time. Lastly, the outcome measure was limited to individual simulation-based assessment which may bias towards the experimental group. Future studies could determine the outcomes of the e-RAPIDs on actual clinical practice."

21) CONSORT: Generalisability (external validity, applicability) of the trial findings

21-i) Generalizability to other populations

No applicable due to limitations described in 20 (I)

21-ii) Discuss if there were elements in the RCT that would be different in a routine application setting

Not applicable

22) CONSORT: Interpretation consistent with results, balancing benefits and harms, and considering other relevant evidence

22-i) Restate study questions and summarize the answers suggested by the data, starting with primary outcomes and process outcomes (use)

"The effectiveness of the e-RAPIDS was evaluated by comparing with mannequin-based simulation. Comparing between these two simulations modalities, we acknowledged that the variations in structures and designs were recognized as confounding variables. It is important to address the confounding variables for the observed results [15]. Using a self-directed learning approach, the RAPIDS training provided learners control of their training agenda, allowing repeated "deliberate practice' and receiving standardized feedback. The mannequin-based simulation utilized a collaborative learning approach that required the learners to perform hands-on simulation in small group and engage group debrief led by a trained facilitator. With this confounding variable, it is crucial to provide evidence to support the validity of the outcome measure [15]. The outcome measure selected for this study was closely aligned with the learning objectives. In both the e-RAPIDS and mannequin-based simulation, the ABCDE and SBAR mnemonics provided the frameworks to guide learning of the training contents. They were also constructs for the RAPIDS evaluation tool which was previously validated and used to measure the performance outcomes during the simulation-based assessment [1]. The validity of the RAPIDS-tool has been well established in a previous study, based on consensus among a panel of clinical experts [13]. "

22-ii) Highlight unanswered new questions, suggest future research

"Future study could evaluate the competence over a longer period of time. Lastly, the outcome measure was limited to individual simulation-based assessment which may bias towards the experimental group. Future studies could determine the outcomes of the e-RAPIDs on actual clinical practice."

Other information

23) CONSORT: Registration number and name of trial registry

This is an educational research, not clinical trial.

24) CONSORT: Where the full trial protocol can be accessed, if available

Available on request from authors

25) CONSORT: Sources of funding and other support (such as supply of drugs), role of funders

This study was funded by a teaching enhancement grant from National University of Singapore Center for Development of Teaching and Learning to Alice Lee Centre for Nursing Studies

X26-i) Comment on ethics committee approval

"The study was approved by the National University of Singapore (NUS) Institutional Human Research Ethics Board."

x26-ii) Outline informed consent procedures

"they were given a participant information sheet that explained the purpose of the study. "

X26-iii) Safety and security procedures

"The students were assured that their decision to participant or not to participate will not affect their training."

X27-i) State the relation of the study team towards the system being evaluated

No conflict of interest has been declared by the authors.
